# Choroidal thickness assessment in keratoconus patients treated with cross-linking compared to healthy population

**DOI:** 10.1007/s10792-022-02517-w

**Published:** 2022-09-23

**Authors:** Antonio Ballesteros-Sánchez, Concepción De-Hita-Cantalejo, María Carmen Sánchez-González, María-José Bautista-Llamas, José-María Sánchez-González, Beatriz Gargallo-Martínez

**Affiliations:** 1 Department of Ophthalmology, Clínica Novovisión, Murcia, Spain; 2 Departament of Physics of Condensed Matter, Optics Area, University of Seville, Seville, Spain; 3 Department of Ophthalmology, Optometry, Otorhinolaryngology and Anatomic Pathology, University of Murcia, Murcia, Spain

**Keywords:** Keratoconus, Choroidal imaging, Optical coherence tomography, Choroidal thickness

## Abstract

**Purpose:**

To analyze the choroidal thickness between patients with keratoconus undergoing cross-linking treatment and a healthy population, as well as to determine the factors that influence choroidal thickness.

**Methods:**

This was an observational, analytical, case–control study that was conducted from February 2021 to June 2021. Choroidal thickness was measured at different locations, including the subfoveal, nasal (1000 μm), temporal (1000 μm), superior (1000 μm) and inferior (1000 μm) locations using a Spectral-domain optical coherence tomography with enhanced depth imaging, which allowed us to obtain horizontal and vertical B-scans centered on the fovea.

**Results:**

This study included 21 patients with keratoconus (mean age, 21.86 ± 5.28 years) and 28 healthy patients (mean age, 24.21 ± 4.71 years). Choroidal thickness was significantly greater in patients with keratoconus than in healthy patients in each of the following measured locations: subfoveal (*P* < 0.001); nasal (1000 μm) (*P* < 0.001), temporal (1000 μm) (*P* < 0.001), superior (1000 μm) (*P* < 0.001) and inferior (1000 μm) (*P* < 0.001) locations. Variables such as age (*ρ* = − 0.09; *P* = 0.50) and refraction (*ρ* = 0.14; *P* = 0.34) were not found to be associated with choroidal thickness. In a stepwise multiple linear regression, the group was the single variable correlated with choroidal thickness (*β* = 0.88; *P* < 0.001).

**Conclusion:**

Choroidal thickness is thicker in keratoconus patients treated with cross-linking than in the healthy population. This finding could be associated with inflammatory choroidal mechanisms in keratoconus patients, but more studies are needed. Age and refractive error do not seem to influence choroidal thickness.

## Introduction

Keratoconus is defined as bilateral and asymmetrical corneal ectasia. It is characterized by progressive corneal protrusion and thinning, which results in its conical shape [1]. Clinically, patients present with irregular astigmatism and myopia with loss of visual acuity even with optimal spectacle correction, thus leading to a considerable impact on the patient´s quality of life [2].

Keratoconus remains one of the main reasons for corneal transplantation [3]. Therefore, the main goal in the treatment of this disorder is to stop the progression of ectasia, which is achieved via cross-linking (CXL). This surgical technique is an effective and safe procedure with minimal complications [4].

Keratoconus has been classically defined as a noninflammatory disease. However, some studies have suggested that inflammation could play a role in the pathogenesis of keratoconus [5, 6]. Increased levels of inflammatory mediators, such as immunoglobulin E (IgE), cytokines (IL-1, IL-6 and TNF-alpha) and proteolytic enzymes (MMP-9), have been found in the tears of keratoconus patients [7, 8]. These inflammatory mediators create a microenvironment that can alter the remodeling process of the extracellular matrix, thus leading to a progressive weakening of the corneal tissue [9, 10]. The choroid is one of the most highly vascularized tissues of the body and is comprised of blood vessels, melanocytes, collagen, fibroblasts, connective tissue and immune cells [11]. The choroidal thickness is approximately 200 mm at birth and can be influenced by the use of drugs [12] and the presence of inflammatory ocular diseases, such as posterior uveitis [13].

Some studies have analyzed choroidal thickness in patients with keratoconus, with the results showing that it is statistically thicker than in healthy populations [14, 15]. However, the correlation between the choroid and keratoconus, which is a degenerative condition defined as being purely corneal, is still unclear. For this reason, we aimed to analyze the choroidal thickness in patients with keratoconus compared to a healthy population.

## Methods

### Patients

This was an observational, analytical, case–control study that was performed at Novovision Ophthalmology Clinic between February and June 2021. It fulfilled all of the requirements of the Declaration of Helsinki and was approved by the research ethics committee of the University of Murcia (CEIC). Prior to initiating the study, informed consent was obtained from each participant.

The inclusion criteria were as follows: ages between 15 and 35 years, a clinical diagnosis of keratoconus and spherical and cylindrical refractive errors less negative than − 8.00 D and less than 1.00 D, respectively (for the control group). Exclusion criteria included the existence of any systemic disease or ocular pathology other than keratoconus, ophthalmological histories of ocular trauma or refractive surgery, systemic or topical uses of vasoactive drugs and keratoconus undergoing CXL treatment with less than 6 months postoperative evolution.

All of the patients underwent a complete ophthalmic examination, which included noncontact tonometry CT 80 (Topcon Corporation, Tokyo, Japan), best-corrected visual acuity (BCVA), slit-lamp examination, corneal topography via Pentacam (Oculus Optikgeräte GmbH, Wetzlar, Germany) and fundus examination via Spectralis Spectral-Domain OCT (Heidelberg Engineering, Heidelberg, Germany). For the statistical analysis, only one eye was randomly selected.

### Spectral-domain OCT scanning: measurement of the choroidal thickness

**Fig. 1:**
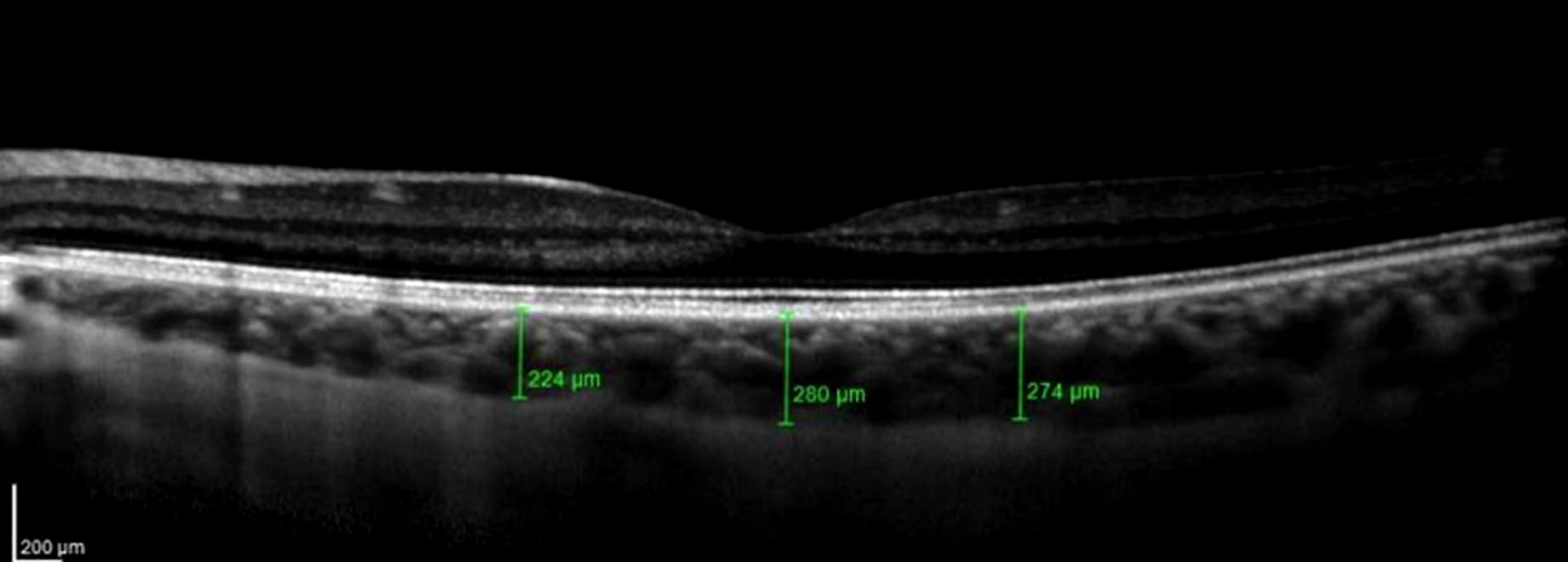
Choroidal thickness measurement with EDI Spectral-Domain OCT. The measurements shown in the image are (from left to right): nasal (1000 μm), Subfoveal and temporal (1000 μm)

Choroidal thickness was determined by using Spectralis Spectral-Domain OCT (Heidelberg Engineering, Heidelberg, Germany) with an enhanced depth imaging (EDI) configuration. The characteristics of the image were horizontal and vertical B-scans centered on the fovea, with the utilization of 100 frames to increase the signal-to-noise ratio. Choroidal thickness was measured manually by the same examiner (ABS) on the fundus image that was obtained with EDI spectral-domain OCT from the outer edge of the retinal pigment epithelium (RPE) to the choroidal-scleral junction (Fig. 1). These measurements were taken at the subfoveal choroid and at 1000 μm intervals from the fovea, including nasal at 1000 μm (N1000), temporal at 1000 μm (T1000), superior at 1000 μm (S1000) and inferior at 1000 μm (I1000). The median of the measurements in the different locations was analyzed to compare the choroidal thickness between the two groups. To reduce the influence of circadian rhythms on choroidal thickness, all measurements were performed from 10 am to 1 pm.

### Statistical analysis

Statistical analyses were performed with SPSS statistics, version 21.0 (IBM Corporation, Armonk, NY, USA). The sample size calculation based on choroidal thickness was evaluated by using the GRANMO calculator, version 7.12 (Municipal Institute of Medical Research, Barcelona, Spain), obtaining a total of 19 patients in both groups. The Shapiro–Wilk test was used to test the normality of the analyzed variables. Continuous variables are displayed as the mean and standard deviation (SD) or median and interquartile range (IQR). The *χ*^2^ test was used to compare the differences in the categorical variables between the 2 groups. The 2-sample *t* test and U Mann–Whitney 2-sample test were used to compare the differences between the continuous variables for those patients who did and did not follow a normal distribution, respectively. Spearman correlation coefficient and multiple linear regression analyses were used to detect the influential factors in subfoveal choroidal thickness. The statistical power of type II error was 0.9 and the level of significance was *P* < 0.05 for all of the comparisons.

## Results

### Participants and descriptive data

A total of 49 patients were included in the analysis, with 21 patients belonging to the study group and 28 patients in the control group. The demographic and refractive characteristics of both groups are depicted in Table 1. The distribution of these variables was found to be normal, and we found no significant difference between them in the keratoconus patients and controls (*P* > 0.05). The topographical characteristics of the study group are shown in Table 2.

**Table 1:** Demographic characteristics of the control subjects and patients with keratoconus

	All participants (*n* = 49)	Keratoconus (*n* = 21)	Control (*n* = 28)
Age (years), mean ± SD 95% CI	23.20 ± 5.0 (21.65–24.75)	21.86 ± 5.28 (19.26–24.26)	24.21 ± 4.71 (22.39–26.04)
*Refraction (D), mean* ± *SD*			
Sphere 95% CI	− 2.48 ± 2.20 (− 3.12 to − 1.85)	− 1.54 ± 2.20 (− 2.53 to − 0.53)	− 3,20 ± 1.95 (− 3.96 to − 2.44)
Cylinder 95% CI	− 1.50 ± 2.10 (− 2.13 to − 0.87)	− 2.86 ± 2.82 (− 4.13 to − 1.57)	− 0,49 ± 0,38 (− 0.63 to − 0.34)
Spherical equivalent 95% CI	− 1.99 ± 1.05 (− 2.99 to − 1.69)	− 2.19 ± 1.20 (− 2.74 to − 1.64)	− 1.84 ± 0.90 (− 2.20 to − 1.49)
*Sex, n (%)*			
*M*	25 (51)	12 (57)	13 (46)
*F*	24 (49)	9 (43)	15 (54)
RE	24 (49)	8 (38)	16 (57)
LE	25 (51)	13 (62)	12 (43)

*SD* standard DESVIATION, *M* male, *F* female, *RE* right eye, *LE* left eye

**Table 2:** Corneal parameters of the keratoconus group obtained by pentacam

	Keratoconus (*n* = 21)
*K*1 (D), mean ± SD	44.60 ± 3.42
*K*2 (D), mean ± SD	47.27 ± 4.04
*K*max (D), mean ± SD	51.79 ± 5.63
TCT (μm), mean ± SD	461.14 ± 45.74
TKC, median (IQR)	1 (1–2)

*K*1, Flat meridian; *K*2, Steep meridian; *K*max, Maximum keratometry; *TCT* thinnest corneal thickness, *TKC* topographic keratoconus classification

### Choroidal thickness in keratoconus and healthy populations

**Fig. 2:**
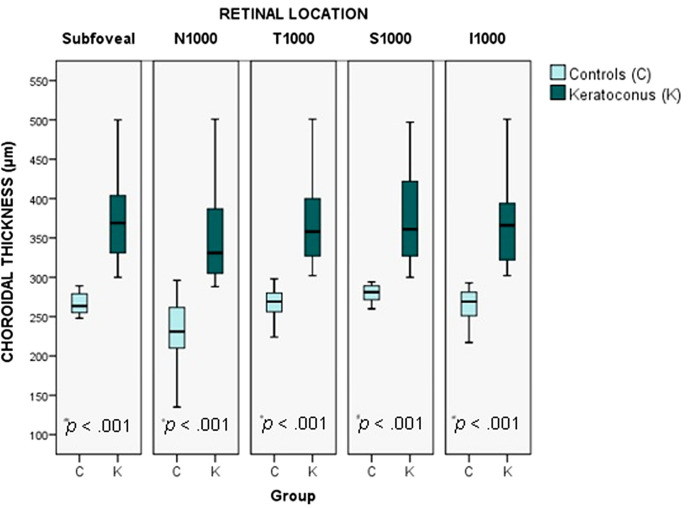
Box and whisker plot (The end of the boxes represent the first and third quartile, the middle line represent the median and the whiskers go from each quartile to the minimum and maximum) of the choroidal thickness in both groups. Choroidal thickness is significant greater in keratoconus eyes than healthy eyes in every measured location. N1000, Measurement undertaken at nasal (1000 μm); T1000, Measurement undertaken at temporal (1000 μm); S1000, Measurement undertaken at superior (1000 μm); I1000, Measurement undertaken at inferior (1000 μm). *P values represent the results of the U Mann–Whitney test

Choroidal thickness did not meet the normality criteria in either group. The comparison of choroidal thickness between the study and control groups is shown in Fig. 2 and Table 3. We found a statistically significant difference in choroidal thickness in every measured location, which was greater in keratoconus patients than in the healthy population (*P* < 0.001).

**Table 3:** Choroidal thickness in both groups

	Keratoconus (*n* = 21)	Control (*n* = 28)	*P* value^*^
Subfoveal (μm), median [IQR]	369 [326–429]	263,50 [255–279]	< 0.001
N1000 (μm), median [IQR]	331 [305–415.50]	231 [210–263.25]	
T1000 (μm), median [IQR]	358 [324–414]	269 [255.50–280.50]	
S1000 (μm), median [IQR]	361 [325.50–433.50]	281 [268.25–289.50]	
I1000 (μm), median [IQR]	366 [319.50–414]	269 [250.50–282]	

N1000, Measurement undertaken at nasal (1000 μm); T1000, Measurement undertaken at temporal (1000 μm); S1000, Measurement undertaken at superior (1000 μm); I1000, Measurement undertaken at inferior (1000 μm)

*P values represent the results of the U Mann–Whitney test

### Influential variables in choroidal thickness.

We analyzed which variables could be associated with choroidal thickness. Choroidal thickness was not found to be associated with age (*ρ* = − 0.09; *P* = 0.50) or refraction (*ρ* = 0.14; *P* = 0.34). In a stepwise multiple linear regression, the group (keratoconus vs control) was the single variable that was correlated with choroidal thickness (95% CI 86.60–164.56; *β* = 0.88; *P* < 0.001). Age (95% CI − 2.64–4.58; *β* = 0.11; *P* = 0.24), sex (95% CI − 51.8–10.38; *β* = 0.09; *P* = 0.33) and refraction (95% CI − 14.74–33.27; *β* = 0.01; *P* = 0.98) did not exhibit statistical significance in the multivariable analysis; thus, they were removed. Therefore, the final model that only included group variables could predict 64% of choroidal thickness (*R* = 0.80; *R*^2^ = 0.64; *P* < 0.001).

## Discussion

In this study, we found that the choroidal thickness in patients with keratoconus was significantly greater than that in the healthy population. We measured choroidal thickness at different locations (subfoveal, nasal [1000 μm], temporal [1000 μm], superior [1000 μm] and inferior [1000 μm]) in both keratoconus and healthy populations by using spectral-domain OCT with EDI configuration. This configuration reduces the light scattering produced by the RPE and the choroidal vessels, thus obtaining a higher quality image of the choroid [16]. We also evaluated the influence of demographic and refractive characteristics on choroidal thickness by using a multiple linear regression analysis. However, the group variable (i.e., the presence or absence of keratoconus) was the single variable that was related to choroidal thickness.

It is known that age has an inversely proportional influence on choroidal thickness [17]. Gutierrez et al. analyzed choroidal vascular density in 52 patients with keratoconus, with a mean age of 43.0 ± 14.0 years, and they determined that there was a progressive decrease in choroidal vascular density with increasing age [13]. We found no statistically significant correlation between age and choroidal thickness (*P* > 0.05). This could be due to the difference in ages between the two studies. The mean age of our study was 23.20 ± 5.0 years, with a minimum age of 15.0 years and a maximum age of 35.0 years.

The influence of refractive error on choroidal thickness is controversial. In a multivariate analysis, Pinheiro-Costa et al. showed that subfoveal choroidal thickness was significantly associated with spherical equivalents (*P* = 0.004) [14]. However, in a recent study, Pinheiro-Costa et al. found that subfoveal choroidal thickness was not associated with spherical equivalents (*P* = 0.079) in a multivariate analysis [18]. The main difference between these studies were the variables that were introduced in the multivariate analysis. These studies have included different variables in the multivariate analyses, which may have affected the influence of refractive error on choroidal thickness. We found that choroidal thickness at the different locations was not associated with refractive error (*P* > 0.05). This could be due to the homogeneity of our sample or to the fact that the refractive range of our sample was not as wide as that of other studies.

In our study, there was a statistically significant difference in choroidal thickness in every measured location between the two groups, which was greater in patients with keratoconus (*P* < 0.001). Some studies have reported similar findings to our results, whereas other studies have found no statistically significant differences in choroidal thickness between keratoconus and healthy patients. In a study that included 45 keratoconus patients with a mean age of 24.5 ± 7.2 years, Akkaya et al. reported that the subfoveal choroidal thickness in keratoconus was significantly greater than that in healthy patients (mean choroidal thickness, 427.48 ± 78.51 μm vs. 351.03 ± 99.08 μm; *P* < 0.001) [19]. Bilgin and Karadag analyzed 80 keratoconus patients with a mean age of 19.0 ± 5.6 years, and they also found statistically significant differences in choroidal thickness between keratoconus and healthy patients (mean choroidal thickness, 363.9 ± 59.8 μm vs. 328.4 ± 67.2 μm; *P* < 0.001) [15]. Pinheiro-Costa et al. also reported similar results as previous studies, in which they found that the subfoveal choroidal thickness was 52.95 μm greater in keratoconus patients (*P* < 0.001) in a study that included 74 keratoconus patients with a mean age of 23.0 ± 4.7 years [14]. However, Yilmaz et al. reported no statistically significant differences in choroidal thickness between keratoconus and healthy patients (*P* > 0.05) [20]. The only difference between this study and the other studies is that it was performed in a pediatric population, with a mean age of 12.2 ± 2.0 years.

The exact pathophysiological mechanism resulting in a thicker choroid in keratoconus patients is not known. Keratoconus has been classically defined as being a noninflammatory disease. However, some studies have suggested that inflammation could play a role in the pathogenesis of the disease [5, 6]. Increased levels of inflammatory mediators such as IgE, cytokines (IL-1, IL-6 and TNF-alpha) and proteolytic enzymes (MMP-9) have been found in the tears of keratoconus patients [7, 8]. These inflammatory mediators create a microenvironment that can alter corneal tissue [9]. This inflammatory process may explain the increased choroidal thickness in patients with keratoconus. The mechanism that produces this increase in choroidal thickness would be the same as that occurring in inflammatory diseases such as Behcet's disease or ankylosing spondylitis, which involves vascular dilatation and stromal infiltration at the level of the choroidal stroma [21, 22]. It is unclear whether this increase in choroidal thickness is an insignificant association in the disease or whether it plays an important role in the pathogenesis of keratoconus, which would make it a potential marker of the disease. Pinheiro-Costa et al. studied whether choroidal thickness was a marker of progression in keratoconus by evaluating choroidal thickness in patients with progressive and nonprogressive keratoconus and found no statistically significant differences between the two groups (*P* = 0.915) [18]. In our study, we did not analyze whether there was a correlation between topographic parameters and choroidal thickness because all of the keratoconus patients were treated with CXL, and keratoconus was stable. CXL influences topographic data, and it is possible that this treatment masks the true influence of topographic characteristics on choroidal thickness.

In the previously mentioned studies, choroidal thickness was analyzed through the use of a horizontal OCT scan, and the study group included patients with keratoconus who were treated or untreated with CXL. However, we analyzed choroidal thickness via horizontal and vertical OCT scans, and all of the keratoconus patients had been previously treated with CXL. Some studies have reported that factors such as age, sex, ethnicity, refractive errors, circadian rhythms, some systemic diseases and the use of vasoactive drugs have an impact on choroidal thickness [23, 24]. However, we tried to reduce the influence of these factors by including patients with similar ages, refractive errors and ethnicity (Caucasian), as well as excluding patients with a systemic disease or the use of vasoactive drugs.

Our study had several limitations. For example, CXL could have influenced choroidal thickness. Some studies have reported retinal changes after CXL without mentioning the choroid [25–27]. Nasrollahi et al. also analyzed the changes in the choroid after CXL, and they demonstrated no statistically significant differences between pre-CXL and post-CXL choroidal thickness at 1 month [28]. In our study, all of the patients were treated with CXL at least 6 months prior to their inclusion in the study, thus reducing the possible influence of CXL on choroidal thickness. The lack of evaluation of axial length was another limitation. Axial length is associated with myopic refractive error and influences choroidal thickness [29]. In this study, only refractive error was measured, which has a good correlation with axial length. The small sample size was also a limitation that may have influenced in our results, but it is important to consider that keratoconus has a low prevalence. Further research is needed to analyze the changes in choroidal thickness in patients with keratoconus, especially in the pediatric population, to better understand whether there is a causal correlation between choroidal thickness and corneal changes and to determine choroidal thickness as a marker of keratoconus progression.

In conclusion, our findings suggest that choroidal thickness is significantly thicker in keratoconus patients treated with CXL than in healthy patients, which could be associated with inflammatory choroidal mechanisms in keratoconus. In contrast to other studies, variables such as age and refractive error do not influence choroidal thickness.
