# Correction: Choroidal thickness assessment in keratoconus patients treated with cross-linking compared to healthy population

**DOI:** 10.1007/s10792-022-02568-z

**Published:** 2022-12-27

**Authors:** Antonio Ballesteros-Sánchez, Concepción De-Hita-Cantalejo, María Carmen Sánchez-González, María-José Bautista-Llamas, José-María Sánchez-González, Beatriz Gargallo-Martínez

**Affiliations:** 1 Department of Ophthalmology, Clínica Novovisión, Murcia, Spain; 2 Departament of Physics of Condensed Matter, Optics Area, University of Seville, Seville, Spain; 3 Department of Ophthalmology, Optometry, Otorhinolaryngology and Anatomic Pathology, University of Murcia, Murcia, Spain

## Correction to: Int Ophthalmol 10.1007/s10792-022-02517-w

The article “Choroidal thickness assessment in keratoconus patients treated with cross-linking compared to healthy population”, written by Antonio Ballesteros-Sánchez, Concepción De-Hita-Cantalejo, María Carmen Sánchez-González, María-José Bautista-Llamas, José-María Sánchez-González, Beatriz Gargallo-Martínez, was originally published electronically on the publisher’s internet portal on 23 September 2022 without open access. With the author(s)’ decision to opt for Open Choice, the copyright of the article changed on 27 October 2022 to © The Author(s) 2022 and the article is forthwith distributed under a Creative Commons Attribution 4.0 International License, which permits use, sharing, adaptation, distribution, and reproduction in any medium or format, as long as you give appropriate credit to the original author(s) and the source, provide a link to the Creative Commons licence, and indicate if changes were made. The images or other third-party material in this article is included in the article’s Creative Commons licence, unless indicated otherwise in a credit line to the material. If material is not included in the article’s Creative Commons licence and your intended use is not permitted by statutory regulation or exceeds the permitted use, you will need to obtain permission directly from the copyright holder. To view a copy of this licence, visit http://creativecommons.org/licenses/by/4.0. Open Access funding provided thanks to the CRUE-CSIC agreement with Springer Nature.

The original article has been corrected.

